# Enacting national social distancing policies corresponds with dramatic reduction in COVID19 infection rates

**DOI:** 10.1371/journal.pone.0236619

**Published:** 2020-07-30

**Authors:** Daniel J. McGrail, Jianli Dai, Kathleen M. McAndrews, Raghu Kalluri

**Affiliations:** 1 Department of Systems Biology, University of Texas MD Anderson Cancer Center, Houston, TX, United States of America; 2 Department of Cancer Biology, Metastasis Research Center, University of Texas MD Anderson Cancer Center, Houston, TX, United States of America; 3 School of Bioengineering, Rice University, Houston, TX, United States of America; 4 Department of Molecular and Cellular Biology, Baylor College of Medicine, Houston, TX, United States of America; University of Waterloo, CANADA

## Abstract

The outbreak the SARS-CoV-2 (CoV-2) virus has resulted in over 6.5 million cases of COVID19, greatly stressing global healthcare infrastructure. Lacking medical prophylactic measures to combat disease spread, many nations have adopted social distancing policies in order to mitigate transmission of CoV-2. While mathematical models have suggested the efficacy of social distancing to curb the spread of CoV-2, there is a lack of systematic studies to quantify the real-world efficacy of these approaches. Here, we first demonstrate that implementation of social distancing policies in US states corresponded with a reduction in COVID19 spread rates, and that the reduction in spread rate is proportional to the average change in mobility. We validate this observation on a worldwide scale by analyzing COVID19 spread rate in 134 nations with varying social distancing policies. Globally, we find that social distancing policies significantly reduced the COVID19 spread rate, with resulting in an estimated 65% reduction (95% CI = 39–80%) in new COVID19 cases over a two week time period. These data suggest that social distancing policies may be a powerful tool to prevent spread of COVID19 in real-world scenarios.

## Introduction

COVID19, caused by the novel coronavirus SARS-CoV-2 (CoV-2) [1], was declared a global pandemic by the World Health Organization on March 11^th^ 2020. As of June 5^th^ 2020, the disease had spread to over 6.5 million cases worldwide, straining the global healthcare infrastructure, and is rapidly becoming a leading cause of death [2]. Medical interventions to address COVID19 are lacking both in the context of prophylactic vaccination approaches, as well as pharmaceutical interventions to treat infected patients. Some countries such as Singapore have demonstrated the feasibility of contact tracing when sufficient testing capacities are available to quarantine exposed individuals and contain disease spread [3]. However, contact tracing may not always be possible to directly implement due to lack of testing availability, lack of infrastructure/funding to perform contact tracing, imprecise data a patient’s interactions while they were contagious, general public compliance, or numerous other obstacles. In nations where contact tracing is not feasible, “social distancing” policies have been implemented by a subset of governments. These policies include closure of non-essential workplaces and schools, as well as policies on physical spacing when in public aimed at preventing viral transmission to reduce the number of new COVID19 cases.

Mathematical models of COVID19 spread have indicated that social distancing policy will likely reduce the spread of CoV-2 [4], with some simulations predicting social distancing policies will reduce COVID19 deaths in the US alone by over half a million [5,6]. Case reports from regions nearby Hubei province where the virus originated have demonstrated social distancing policies will reduce viral transmission [7], as well as through China [8]. However the efficacy of the approach has yet to be evaluated on a global scale. Here, using data from the 50 US States and 134 nations we assess the efficacy of social distancing and find that social distancing significantly prevents spread of COVID19, resulting in an estimated 65% reduction in new COVID19 cases globally in countries that implemented national social distancing policies.

## Methods

### Data sources

All data were acquired on June 5^th^ 2020. Daily case numbers for COVID19 and population numbers were acquired from the COVID-19 Data Repository by the Center for Systems Science and Engineering (CSSE) at Johns Hopkins University (https://github.com/CSSEGISandData/COVID-19). Social distancing policies were acquired from https://auravision.ai/covid19-lockdown-tracker/. Mobility data were acquired from Google mobility reports (https://www.google.com/covid19/mobility/). Average mobility was taken as the average reduction in mobility across the 5 Google mobility metrics (retail and recreation, grocery and pharmacy, parks, transit stations, and workplace). For regions with multiple sub-region values, all values were averaged. Testing data was acquired from the Our World In Data source data repository (https://github.com/owid/covid-19-data/tree/master/public/data/). Countries were considered for inclusion if they had over 1 case per million of COVID19, and at least 14 days of data prior to and 21 days of data following implementation of social distancing policy (or equivalent time period in countries without a social distancing policy). Countries with regional social distancing policies were excluded from analysis. Tabulation of relevant parameters and additional notes can be found in S1 Table.

### Data analysis

All data analysis was performed in MATLAB R2019a. US heat map of COVID19 spread rate was generated in R v3.6 using the packages “maps” and “ggplot2”. For per country or state COVID19 spread rates, data were modeled to an exponential growth equation *Cases* ~ *Cases0*∙exp(*k*_spread_∙*t*), where Cases is cases per million inhabitants, Cases0 is the number of cases per million inhabitants at the initial time point, k_spread_ is the COVID19 spread rate, and t is the time point in days. Patients who recovered or died were subtracted from total number of cases. Unless otherwise specified, we used the following parameters for analysis (see **S1 Fig**). The initial time point was considered as when countries exceeded 1 case per million inhabitants. Post social distancing was considered 11 days after social distancing policy was enacted based on disease latency days [9,10]. For countries with no social distancing policies, we used the median time interval from reaching 1 case per million to enacting social distancing policies in states/countries analyzed with social distancing policies, corresponding to 16 days in US States and 17 days globally. Models were fit out to 21 days for each time period. Based on the 95% confidence interval for time from exposure to exhibiting symptoms of 8.2–15.6 days [10], we allowed a maximum of 7 days into initiation of social distancing policies to still be considered pre-social distancing as any new infections most likely would have occurred before policy implementation. For additional global modeling of COVID19 spread, the above equation was utilized in a generalized linear mixed-effects model taking each country as a random effect. To estimate prevented number of cases, the change in COVID19 spread rate in countries with social distancing policies was equated to that of those without social distancing. Final total number cases was determined after 14 days of spread.

### Statistics

For comparison of groups was made using either a either a rank-sum test (two groups) or Kruskal-Wallis with Dunn’s posthoc test (more than two groups). Correlations were assessed with Spearman correlation coefficient. Paired analysis was performed using a signed-rank test.

## Results

### Statewide social distancing policies in the United States correspond with reduced COVID19 spread rate proportional to reductions in mobility

**Fig 1:**
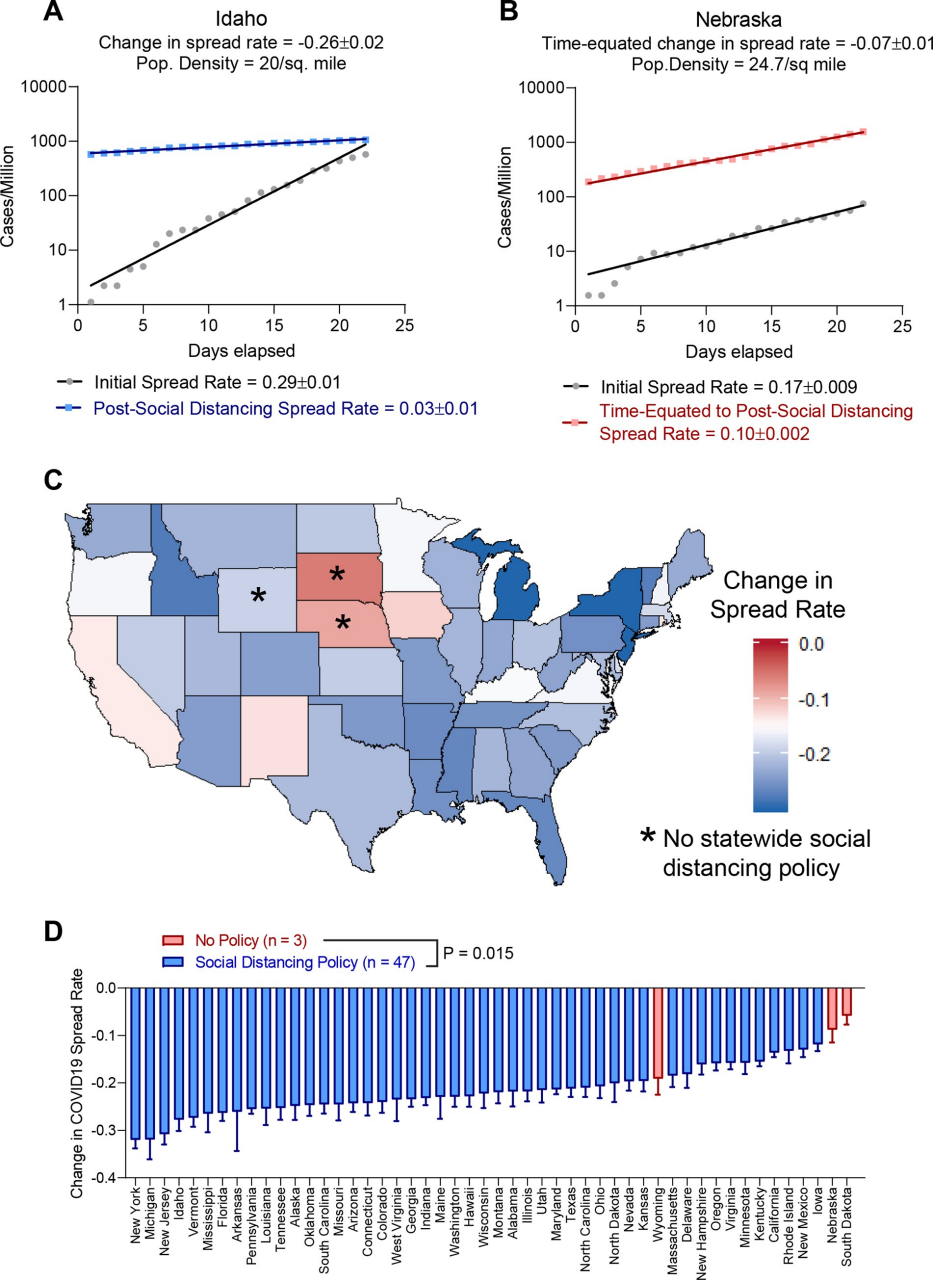
Social distancing policies corresponded with inhibited spread of COVID19 in the United States. **(A)** Fitting of COVID19 initial spread rate and spread rate following social distancing implementation in Idaho. (**B**) Fitting of COVID19 initial spread rate and a second time interval matched to post-social distancing in Nebraska where no policy was implemented. **(C)** Heat map showing change in COVID19 spread rate after implementing statewide social distancing policy, or a time-matched interval in states without social distancing policies (Nebraska, Wyoming, and South Dakota). **(D)** Bar graph showing values in (C), with states that did not implement social distancing policies shown in red. Error bars indicate 95% confidence interval. Rank-sum test.

In order to assess the efficacy of social distancing policies in the United States, we began by quantifying the COVID19 spread rate as defined by an exponential growth function both before and after social distancing policies were enacted. Analysis of COVID19 spread rates may be complicated by numerous features, including population demographics and densities, travel rates, variations in testing accessibility, weather patterns, and likely many more parameters yet to be identified. To account for these factors, we determined the spread of COVID19 both before and after implementation of social distancing policies. As features influencing COVID19 spread rate may also vary over time, we additionally analyzed the 3 US states (Nebraska, Wyoming, and South Dakota) that did not implement social distancing policies. For these 3 states without social distancing policies, we used the median time that other states took to enact social distancing policies **(S1B Fig)**. This approach is illustrated by comparison of Idaho and Nebraska, two states with similar population densities **(Fig 1A and 1B)**. Idaho enacted social distancing policies on March 25^th^ 2020, whereas no such policies were enacted in Nebraska. In Idaho, pre-social distancing COVID19 spread rate was 0.29, dropping to 0.03 following social distancing for a net change of -0.26 **(Fig 1A)**. When analyzing spread rate in Nebraska over equivalent time periods we found that Nebraska only dropped from 0.17 to 0.10, for a net change of -0.07, 3-fold lower than observed in Idaho **(Fig 1B)**. Results for the entire United States are shown in **Fig 1C and 1D**. Notably, the two smallest reductions in COVID19 spread rates were observed in states without social distancing policies (South Dakota and Nebraska), with Wyoming having the 15^th^ smallest reduction.

**Fig 2:**
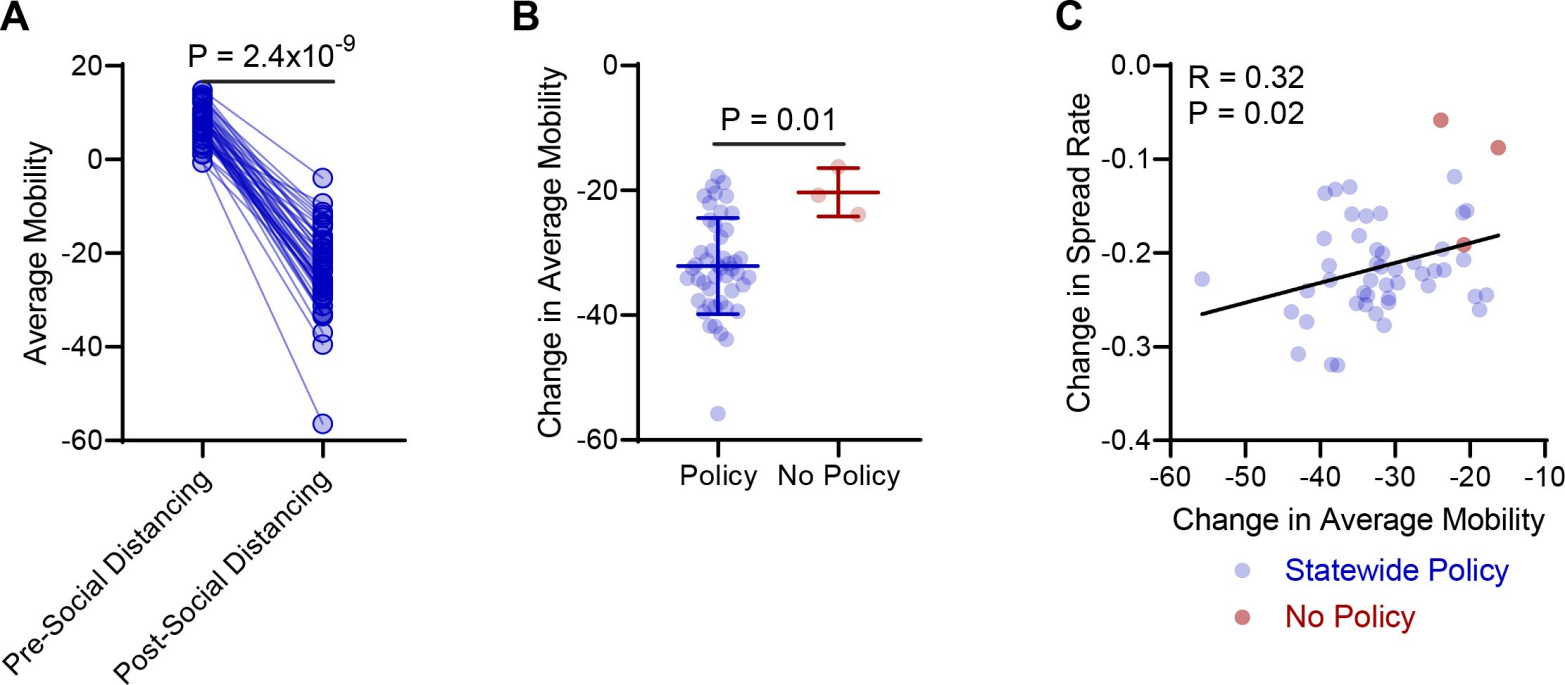
Social distancing policies correspond with reduced average mobility in the United States. **(A)** Average mobility from Google mobility reports prior to implementation of social distancing and after implementation of social distancing in US states that enacted social distancing policies. Signed rank test. **(B)** Change in average mobility determined from Google mobility reports following implementation of social distancing policies (blue), or time-equated periods in states with no social distancing policies (red). Median with interquartile range. Rank-sum test. **(C)** Correlation of change in COVID19 spread rate and change in average mobility. Spearman correlation coefficient.

We next investigated the effect of social distancing policies on community mobility. In the 47 states with social distancing policies we observed a strong reduction in average mobility following implementation of social distancing policies (P = 2.4x10^-9^, **Fig 2A**). While states without social distancing policies also showed decreased mobility over the same time frame, the observed decrease was significantly less, suggesting social distancing policies effectively reduce community mobility **(Fig 2B)**. Critically, changes in average mobility were significantly correlated with decreases in COVID19 spread rate **(Fig 2C)**.

### Global reduction in COVID19 spread rates associated with social distancing policies and reduced mobility

**Fig 3:**
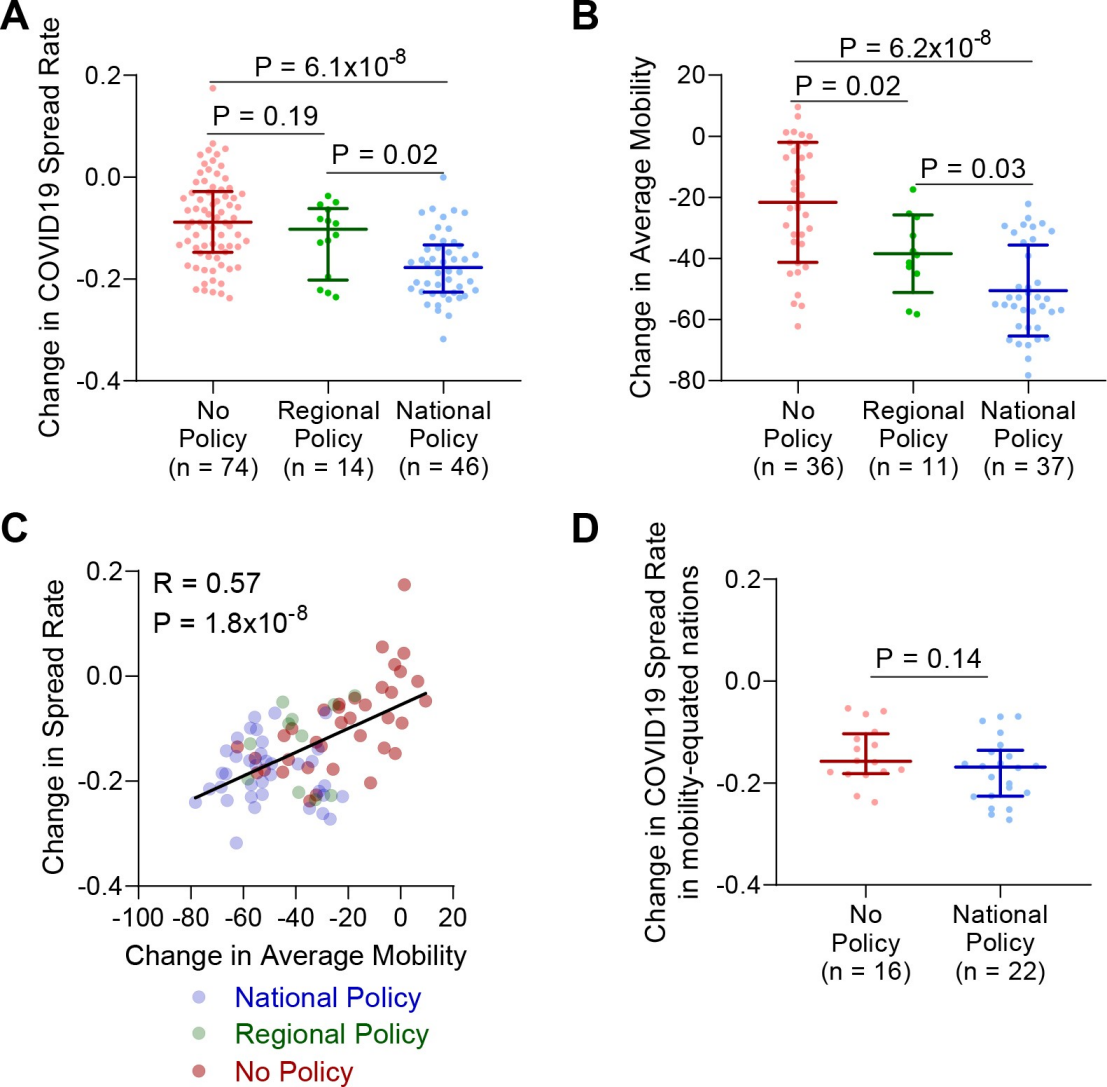
Global reduction of COVID19 spread reduced mobility through social distancing policies. **(A)** Change in COVID19 spread rate following implementation of social distancing policy, or time matched interval in countries with no policy. Kruskal-Wallis test with Dunn’s posthoc. **(B)** Change in average mobility from Google mobility reports following implementation of social distancing policy, or time matched interval in countries with no policy. Kruskal-Wallis test with Dunn’s posthoc. **(C)** Correlation of change in COVID19 spread rate and change in average mobility. N = 84 countries. Spearman correlation coefficient. **(D)** Analysis of change in COVID19 spread rates per (A) only in nations with overlapping changes in average mobility, defined as from -56 to -22. Rank-sum test.

While analysis of US states indicated that social distancing policies reduced COVID19 spread rates proportional to associated reductions in mobility, only having 3 states without social distancing policies reduces the power of any observations. To address this, we next expanded our model to the global level. In total, there were 74 nations with no social distancing policy, 14 nations with a regional social distancing policy, and 46 nations with a national social distancing policy that had sufficient data for analysis. Following implementation of a national social distancing policy, nations observed a larger decrease in COVID19 spread rate than nations with regional social distancing policies, or the change over an equivalent time frame in nations without a social distancing program **(Fig 3A)**. To further validate this observation we repeated this analysis using a variety of different time periods and found all models robustly recapitulated the reduction in COVID19 cases observed in nations with national social distancing policies **(S2 Fig)**. Likewise, analysis of both testing rates in the pre-social distancing time period and the change in testing rates showed no significant difference between countries with and without national policies, although countries with regional policies displayed lower pre-social distancing testing rates **(S3A–S3C Fig)**. The reduction in COVID19 spread rates with national social distancing policies was also conserved when equating for initial COVID19 spread rates **(S3D Fig)**. No difference in reduction of COVID19 spread rates was observed between nations without a social distancing policy and those with a regional policy. As observed in US states, nearly all countries demonstrated reductions in mobility over this time period with the exception of Mongolia, Guinea-Bissau, Nicaragua, Uganda, and Tanzania. Nations with regional or national social distancing policies exhibited a significantly larger reduction in mobility than those without policies, and nations with national policies exhibited a significantly larger reduction than those with regional policies **(Fig 3B)**. Correlation between the change in COVID19 spread rate and change in mobility in 84 nations across the globe indicated a strong correlation between decreased COVID19 spreading and decreased mobility **(Fig 3C)**. A subset of 16 nations without social distancing policies exhibited reductions in mobility equivalent to those observed in nations with social distancing policies. Upon comparing only nations with overlapping changes in average mobility, that is greater than -56% and less than -22%, we no longer observed a significant difference in change in COVID19 spread rates between countries with and without social distancing policies **(Fig 3D)**.

**Fig 4:**
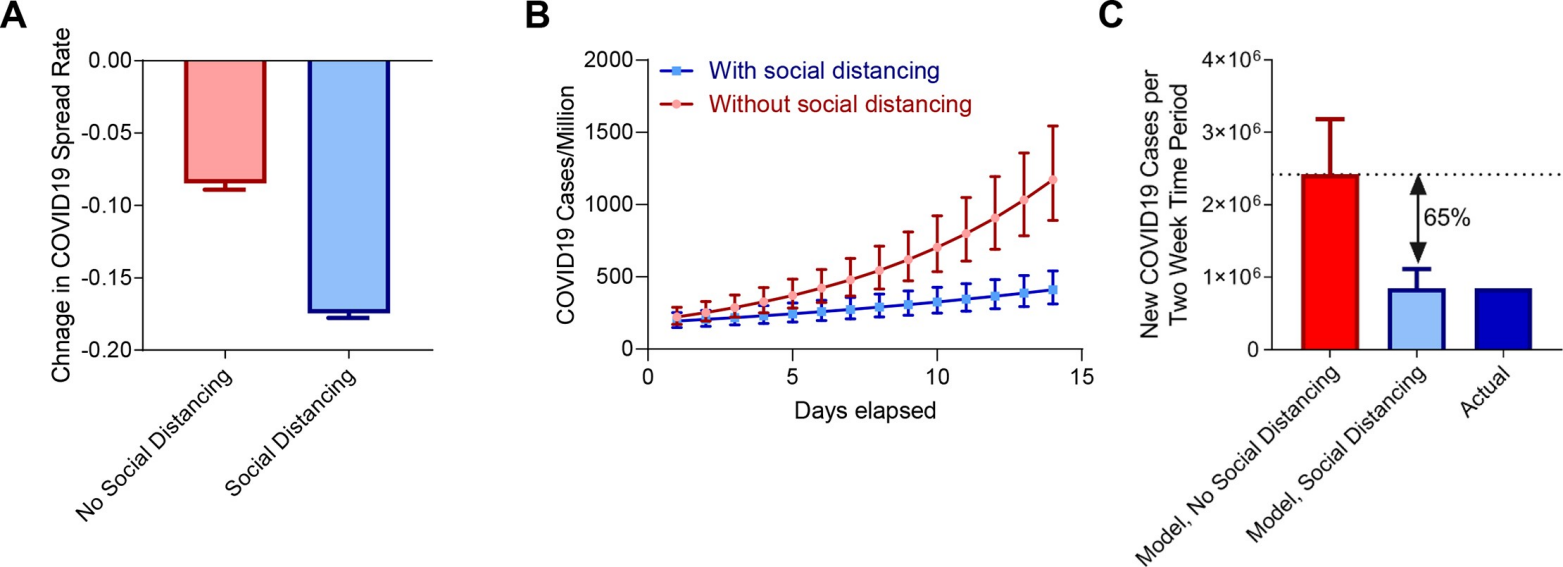
Prevention of new COVID19 cases by social distancing policies. **(A)** Change in COVID19 spread rates following implementation of social distancing policies (blue), or time-equated periods in countries with no social distancing policies (red). Data were fit using a generalized linear mixed-effects model taking each country as a random effect. Bars represent change in regression coefficient ± standard error. **(B)** Modeling of new COVID19 cases per million inhabitants in countries with social distancing policies using either model fit directly to data following implementation of social distancing policies (blue), or after correcting for the observed reduction in COVID19 spread rates associated with social distancing policies (red). Points represent model COVID19 cases per million ± standard error. **(C)** Estimation of total new cases over a two-week period if countries had not implemented social distancing policies (red), with implementation of social distancing policies (light blue), and actual new cases (dark blue). Bars represent number of COVID19 cases in the countries with social distancing policies ± standard error.

In order to assess the benefit of this reduction in COVID19 spread rates, we refined our calculations using generalized linear mixed effect models taking each country as a random effect. This approach again recapitulated the larger drop in COVID19 spread rates observed in countries with social distancing policies (**Fig 4A**). To quantify the healthcare benefits resulting from this reduction in spread rate, we calculated COVID19 spread rates in countries with social distancing policies both using the model directly fit to the data, as well as correcting for the reduction in COVID19 spread rate observed following implementation of social distancing policies (**Fig 4B**). After accounting for number of inhabitants, this approach indicates that over a two week period social distancing prevented an estimated 1.57 million cases of COVID19 in the 46 nations with social distancing policies, representing a 65% reduction (95% CI = 39–80%) in new COVID19 cases (**Fig 4C**).

## Discussion

Our analysis of COVID19 spread rates in 134 countries quantifies the effects of social distancing at preventing CoV-2 transmission. A critical limitation of this study is reliance on direct COVID19 testing, which likely underestimate prevalence when compared to antibody-based serology testing approaches [11]. Numerous additional factors such as population density, healthcare infrastructure, testing rates, climate, population characteristics, and more, likely contribute to rate of COVID19 spread, this study focused on the change in COVID19 spread rate following implementation of social distancing policies as an internal control for these variables. This analysis found that social distancing policies significantly reduced spreading rates compared to time-matched intervals in nations that did not implement social distancing policies. Although social distancing policies may have negative economic impacts [12], this analysis suggests that this containment approach has yielded significant positive health outcomes. Despite the acute economic depression associated social distancing policies, long-term estimates indicate the prevention of mortality in the US could save $8 trillion dollars nationally, or $60,000 per US household [6]. Refinement of COVID19 epidemiological models will be critical to assist policy makers in deciding when and how to lift social distancing policies.

While here we only considered countries with a national/regional social distancing policy, some nations without a technical policy have implemented measures to slow COVID19 spread. For instance, South Korea has not enacted a social distancing program but instead utilized a powerful contact tracing approach to control the spread of COVID19. In our analysis, we found South Korea had the 25^th^ largest (out of 134) reduction in COVID19 spread rate, indicating inclusion as a “no policy” country will suppress the estimate of benefits of social distancing. Although this classification may be imperfect, potential improper assignment of countries to “no policy” will return a lower bound of the effect of social distancing. Likewise, it is also possible that mobility will be decreased in absence of official social distancing policies, or due to reduced travel from social distancing policies in neighboring areas. Consistent with this, we observed a strong relationship between changes in mobility and changes in COVID19 spread rate. Moreover, when equating for changes in mobility we no longer detected a significant difference in COVID19 spread rate between nations with and without social distancing policies. This observation demonstrates that adherence to social distancing policies is critical to their efficacy, and that some nations were able to achieve reduced mobility without official policies. Understanding sociological factors impacting reduced mobility in absence of government policy may be beneficial for preventing the spread of COVID19.

As testing capacities increase, contact tracing may provide an alternative to minimize economic impacts by only quarantining those who are exposed to infected individuals. This approach has already demonstrated high efficacy in the context of nations with high testing capacity [3]. Technological advancements, such as potential phone-based contact tracing Apps, may further enhance the benefit achieved by contact tracing approaches [13].

The emergence of further refined data will allow for more precise quantification of various approaches to mitigate the spread of COVID19. This analysis based on 134 countries indicates that social distancing can serve as a critical preventative measure until prophylactic measures and larger population immunity are generated.

## Supporting information

S1 Table Countries and states utilized in this study. (XLSX)

S1 Fig Time periods used for data analysis. **(A)** Schematic showing time frames used for data analysis. **(B)** Time from reaching 1 case per million to implementation of social distancing policy in US states where policies were implemented. The median time of 16 days was used for states with no social distancing policy. Median with interquartile range. **(C)** Time from reaching 1 case per million to implementation of social distancing policy in countries where policies were implemented. The median time of 17 days was used for countries with no social distancing policy. Median with interquartile range.
(PDF)

S2 Fig Altering time periods analyzed for modeling minimally impacts results. **(A)** Standard model. **(B)** Increasing the offset time after implementation of social distancing policies to 14 days. **(C)** Enforcing all countries have 21 days of data prior to implementation of social distancing polices. **(D)** Reducing modeling time period to 14 days for all countries. All data shown as median with interquartile range, Kruskal-Wallis with Dunn’s posthoc test.
(PDF)

S3 Fig Testing rates are not altered between countries with social distancing policies and no policy. **(A)** Tests per thousand inhabitants during initial time period before implantation of social distancing policies. **(B)** Rate at which countries increased testing capacity, fit as *Tests* ~ *Tests0*·exp(*k*testing·*t*). **(C)** Difference in testing rate before and after implementation of social distancing policies. All data shown as median with interquartile range, Kruskal-Wallis with Dunn’s posthoc test. **(D)** Change in COVID19 spread rate following social distancing policy implementation (or matched time period) in countries with equated initial COVID19 spread rates.
(PDF)
