# Supplementary material for: Enacting national social distancing policies corresponds with dramatic reduction in COVID19 infection rates
Source: PLoS One. 2020 Jul 30;15(7):e0236619. doi: 10.1371/journal.pone.0236619 (PMC7392246; doi:10.1371/journal.pone.0236619)
Supplement: S1 Fig — (A) Schematic showing time frames used for data analysis. (B) Time from reaching 1 case per million to implementation of social distancing policy in US states where policies were implemented. The median time of 16 days was used for states with no social distancing policy. Median with interquartile range. (C) Time from reaching 1 case per million to implementation of social distancing policy in countries where policies were implemented. The median time of 17 days was used for countries with no social distancing policy. Median with interquartile range. (PDF) [file pone.0236619.s002.pdf]

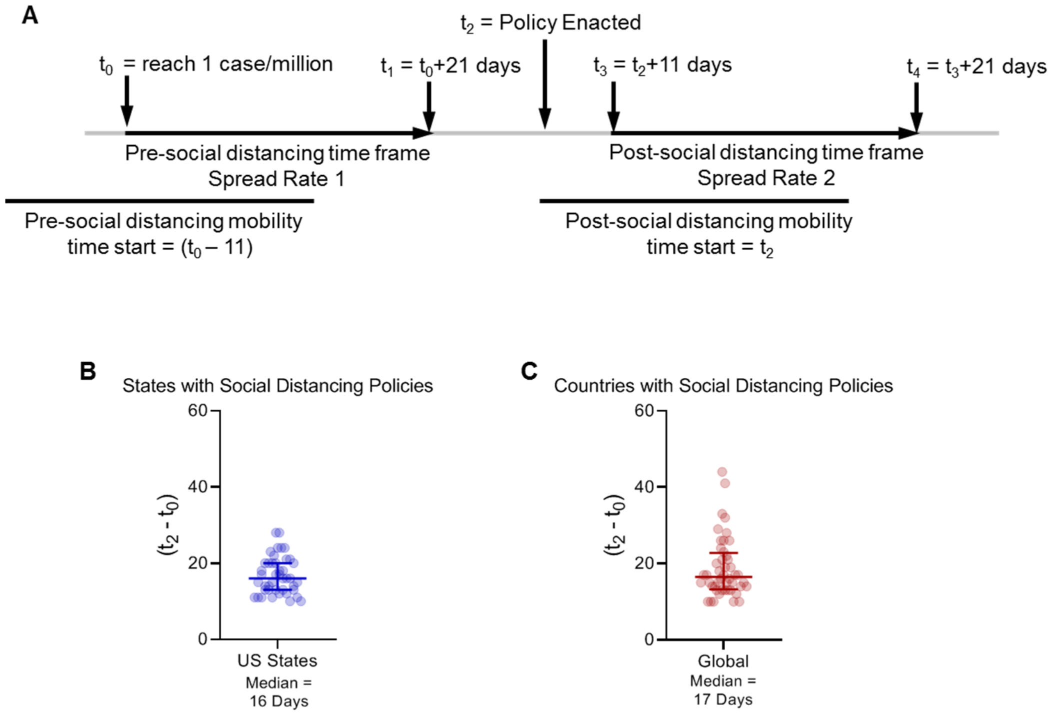

Figure S1. Time periods used for data analysis. (A) Schematic showing time frames used for data analysis. **(B)** Time from reaching 1 case per million to implementation of social distancing policy in US states where policies were implemented. The median time of 16 days was used for states with no social distancing policy. Median with interquartile range. **(C)** Time from reaching 1 case per million to implementation of social distancing policy in countries where policies were implemented. The median time of 17 days was used for countries with no social distancing policy. Median with interquartile range.
