# Supplementary material for: Enacting national social distancing policies corresponds with dramatic reduction in COVID19 infection rates
Source: PLoS One. 2020 Jul 30;15(7):e0236619. doi: 10.1371/journal.pone.0236619 (PMC7392246; doi:10.1371/journal.pone.0236619)
Supplement: S2 Fig — (A) Standard model. (B) Increasing the offset time after implementation of social distancing policies to 14 days. (C) Enforcing all countries have 21 days of data prior to implementation of social distancing polices. (D) Reducing modeling time period to 14 days for all countries. All data shown as median with interquartile range, Kruskal-Wallis with Dunn’s posthoc test. (PDF) [file pone.0236619.s003.pdf]

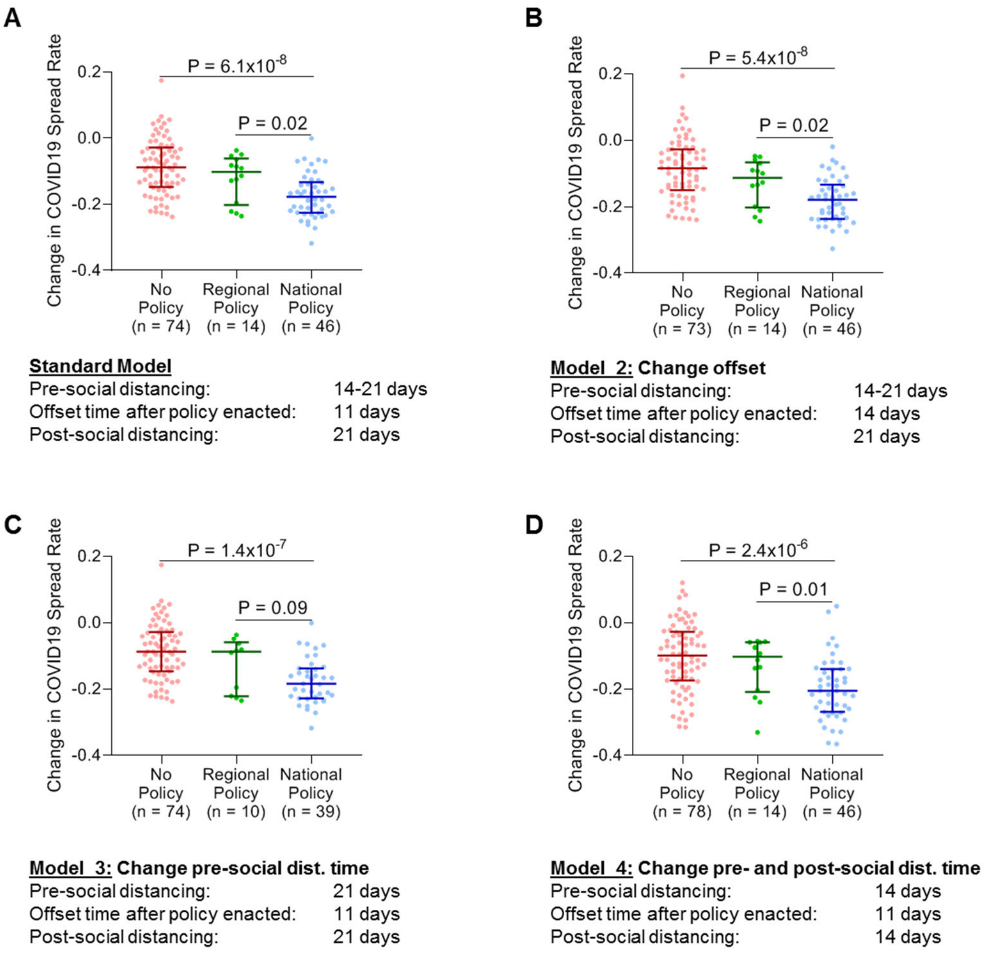

Figure S2. Altering time periods analyzed for modeling minimally impacts results. (A) Standard model. **(B)** Increasing the offset time after implementation of social distancing policies to 14 days. **(C)** Enforcing all countries have 21 days of data prior to implementation of social distancing polices. **(D)** Reducing modeling time period to 14 days for all countries. All data shown as median with interquartile range, Kruskal-Wallis with Dunn's posthoc test.
