# Supplementary material for: Enacting national social distancing policies corresponds with dramatic reduction in COVID19 infection rates
Source: PLoS One. 2020 Jul 30;15(7):e0236619. doi: 10.1371/journal.pone.0236619 (PMC7392246; doi:10.1371/journal.pone.0236619)
Supplement: S3 Fig — (A) Tests per thousand inhabitants during initial time period before implantation of social distancing policies. (B) Rate at which countries increased testing capacity, fit as Tests ~ Tests0·exp(ktesting·t). (C) Difference in testing rate before and after implementation of social distancing policies. All data shown as median with interquartile range, Kruskal-Wallis with Dunn’s posthoc test. (D) Change in COVID19 spread rate following social distancing policy implementation (or matched time period) in countries with equated initial COVID19 spread rates. (PDF) [file pone.0236619.s004.pdf]

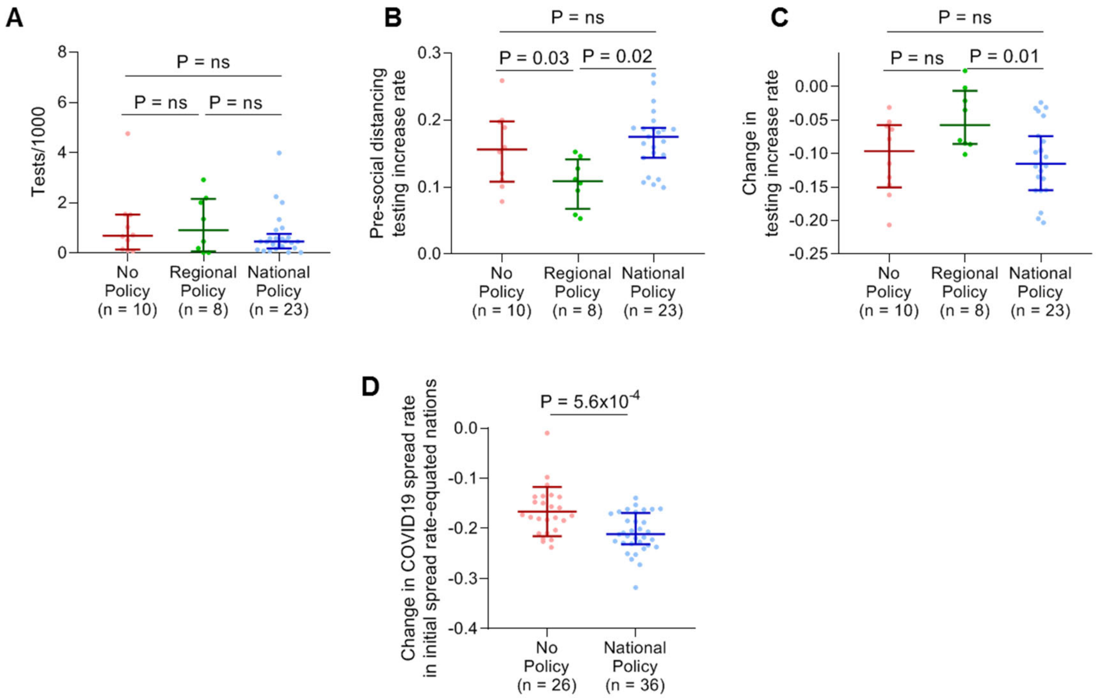

Figure S3. Testing rates are not altered between countries with social distancing policies and no policy. (A) Tests per thousand inhabitants during initial time period before implantation of social distancing policies. **(B)** Rate at which countries increased testing capacity, fit as $Tests \sim Tests0 \cdot \exp(k_{testing} \cdot t)$. **(C)** Difference in testing rate before and after implementation of social distancing policies. All data shown as median with interquartile range, Kruskal-Wallis with Dunn's posthoc test. **(D)** Change in COVID19 spread rate following social distancing policy implementation (or matched time period) in countries with equated initial COVID19 spread rates.
